# Neoplasia detection in FIT positive screening colonoscopies compared with an age-controlled symptomatic cohort: a retrospective review

**DOI:** 10.3389/fgstr.2024.1372191

**Published:** 2024-03-22

**Authors:** Neil O’Morain, Roisin Stack, Jayne Doherty, Blathnaid Nolan, Parker Girod, Lakshman Kumar, Mark McCrossan, Elaine Joy, Orlaith Casey, Gareth Horgan, Glen Doherty

**Affiliations:** ^1^ Centre for Colorectal Disease, St. Vincent’s University Hospital, Dublin, Ireland; ^2^ School of Medicine, University College Dublin, Dublin, Ireland

**Keywords:** colorectal cancer, colorectal cancer screening, fit, lower gastrointestinal symptoms, high risk findings

## Abstract

Colonoscopy following a positive FIT test in an average risk population is effective in reducing CRC incidence and mortality. While lower gastrointestinal symptoms remain a common cause for referral for colonoscopy, symptoms are poor predictors of clinically significant disease. The study was performed to compare neoplasia detection FIT +ve individuals and age-matched symptomatic cohorts. A single centre retrospective observational study was performed including all index colonoscopies performed on patients aged 60-70 from January 2015 to September 2021. Diagnostic yield was reported as adenoma detection rate, SSL detection rate, detection of high risk finding or adenocarcinoma. 8,106 colonoscopies were performed on patients aged 60-70 years. 3,695 (45.6%) originated from screening (FIT +ve). With exclusion criteria applied, 2,640 (59.9%) for screening and 1,767 (40.1%) for symptomatic patients were included. Median age in screening was 65 years (IQR 62-67) and 64 years in the symptomatic group (IQR 62-68), with male predominance in both groups (n=1,536, 58.1%, n=944, 53.4%). There were significant differences in both the ADR (56% vs 26.3%, p<0.01) and the SSLDR (10.4% vs. 8.1%, p=0.05) in the screening cohort compared to the symptomatic group. High risk findings (21.3% vs. 7.5%, p<0.01) were significantly more prevalent in the screening group with a considerably higher colorectal cancer (4.7% vs. 0.9%, p=<0.001) detection rate. FIT based triage significantly outperforms symptom based investigation for individuals in the 60-70 age group. Patients should be preferentially referred to organised colorectal cancer screening. FIT can be performed on symptomatic patients, to identify low risk individuals.

## Introduction

Colorectal cancer (CRC) remains a major cause of mortality and morbidity worldwide. CRC is the third most commonly diagnosed cancer in men and the second most commonly diagnosed cancer in women. It is the second leading cause of cancer related death in men and the third leading cause of death in women, related to its advanced stage when becoming clinically overt. There were over 1.9 million cases of CRC reported globally in 2020, with this figure projected to increase further (1). The estimated incidence of CRC in Ireland is 91.2/100K in men and 60.3/100K in women, with an alarming increase in incidence in younger adults (<50 years) reported in the past decade (2). The estimated mortality in men is 40.8/100K, which is one of the highest reported incidence rates in Europe, and 25.3/100K in women (3). Survival rates for CRC in Ireland have been increased over the past decade, with the majority of screen detected cancers discovered at an early stage (4). There is a significant economic burden associated with CRC including non-healthcare costs related to loss of productivity due to disability and premature death as well as the substantial healthcare costs involved in the treatment of advanced disease (5).

There are currently two main pathways of referral for colonoscopy in Ireland; an organised population based screening programme (BowelScreen) targeting the 59-69 year age group and a symptomatic pathway whereby patients presenting to their General Practitioner are referred to tertiary care (to a Gastroenterologist or Colorectal Surgeon) for further investigation. The stated primary goal of BowelScreen, which commenced in 2014, is to reduce the incidence and mortality of colorectal cancer in people aged 55-74 in Ireland. However, almost a decade later the programme has not expanded significantly beyond the initial limited age range (60-69y), thereby limiting the maximum potential benefit of the screening programme. Furthermore, this does not acknowledge the rising incidence of early-onset CRC. One of the main issues limiting the programme’s expansion is colonoscopy capacity in Ireland, with most endoscopy units in Ireland oversubscribed and breaching national standards for both urgent and routine procedure wait times. Moreover, colorectal cancer screening is only performed in units certified by the Joint Advisory Group on Gastrointestinal Endoscopy (JAG), and by endoscopists accredited by the National Cancer Screening Service who meet specific performance quality indicators (i.e. adenoma detection rate >45%) with 300 colonoscopies performed within a year.

Lower gastrointestinal symptoms remain a common cause for referral for colonoscopy despite the increasing evidence that symptoms alone, or symptom-based triaging systems, are poor predictors of clinically significant disease (6, 7). Indeed, symptoms alone have been shown to have a poor positive predictive value (PPV) for colorectal cancer of only 1-2% (8), with previous studies demonstrating higher rates of neoplasia detection in screening colonoscopies compared to symptomatic patients in younger cohorts (9, 10).

Lack of public awareness, poor uptake and a limited scope of the colorectal cancer screening programme coupled with an over-reliance on symptom based investigation has resulted in an inefficient use of colonoscopy services in Ireland. An initial assessment of this symptomatic cohort with non-invasive diagnostic tools may be more appropriate to exclude clinically significant disease and therefore avoid unnecessary colonoscopy (11, 12).

The aim of this study was to evaluate the diagnostic yield of advanced colorectal polyps and colorectal cancer in index colonoscopies in a colorectal cancer screening cohort compared with an age-controlled symptomatic cohort. The primary outcome was the presence of high risk findings (adenoma +/- high grade dysplasia ≥10mm or serrated lesion +/- dysplasia ≥10mm, or ≥5 premalignant polyps) and/or the presence of adenocarcinoma. The secondary outcome was to determine the positive predictive value of lower gastrointestinal symptoms for neoplasia detection.

## Methods

### Patient selection

A single centre age-controlled retrospective observational study was performed including all index colonoscopies performed on screening and symptomatic patients aged 60-70 from January 2015 to September 2021, corresponding to the first 7 years of the BowelScreen programme. Following research ethics committee approval, screening participants were identified from a prospectively maintained BowelScreen database, and symptomatic patients were identified through interrogation of our electronic endoscopy reporting system. LGIS included for analysis included abdominal pain, change in bowel habit, diarrhoea, constipation, weight loss and PR bleeding. For the purpose of comparison within this study, colonoscopies to investigate anaemia were included in the ‘symptomatic’ group. Surveillance colonoscopies in the screening group were excluded. Colonoscopies performed for indications other than the investigation of lower gastrointestinal symptoms (LGIS) were excluded (e.g. previous CRC/polyps, IBD assessment, diverticular disease, abnormal radiology). The FIT cut off employed by the BowelScreen programme was 45ugHb/gF (equivalent to 225ngHb/ml buffer).

### Data collection

Demographic data including age and gender was recorded. Details of polyp findings at endoscopy including number, size, location were documented. Histopathological findings including polyp type (hyperplastic, adenoma, serrated) and the presence of high-risk findings and colorectal cancer were recorded. The diagnostic yield was reported as the adenoma detection rate (ADR), SSL detection rate (SSLDR), detection of high risk finding, or adenocarcinoma. Quality measures including adequacy of bowel preparation, comfort scores and use of hyoscine butylbromide were recorded. The prevalence of adenomas, serrated, and advanced lesions was reported according to individual symptoms. Positive predictive values colorectal cancer were calculated for the relevant symptoms.

### Statistical analysis

Statistical analyses were undertaken using SPSS version 27 (IBM). Participants with missing data were excluded. All tests were two-tailed and p-values <0.05 were considered significant. Continuous data were reported as median and interquartile ranges (IQR), discrete data as numbers and percentages, unless otherwise stated. Descriptive statistics were used to calculate measures of central tendency in the form of medians for continuous variables (age) and proportions for categorical variables (gender). Univariate analyses, using Mann-Whitney U and Kruskal-Wallis non-parametric test were used to compare continuous variables in the screening and symptomatic cohorts. PPVs were calculated by dividing the number of symptomatic individuals diagnosed with CRC by the total number of symptomatic individuals in each category. The PVs are presented as percentages.

## Results

### Demographic data

A total of 8,106 colonoscopies were performed on patients aged 60-70 years at our institution during the study period. Of these, 3,695 (45.6%) originated from the screening pathway (FIT +ve) with 4,411 (54.4%) performed as non-screening colonoscopies. When surveillance and repeat colonoscopies, as well as those performed for indications other than the investigation of LGIS were excluded, a total of 4,407 index, or first, colonoscopies remained, of which 2,640 (59.9%) were screening colonoscopies and 1,767 (40.1%) were symptomatic.

**Figure 1:**
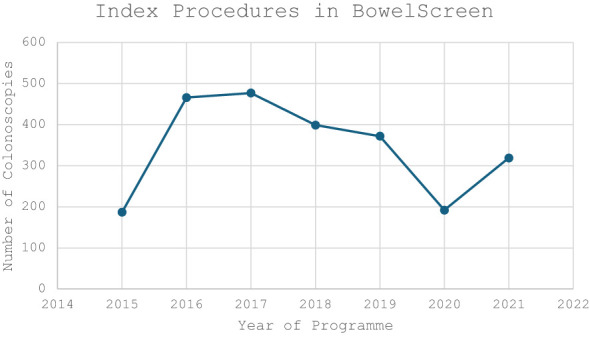
Volume of NCSS BowelScreen Index Colonoscopies performed in our institute during the study period (2015-2021).

**Figure 2:**
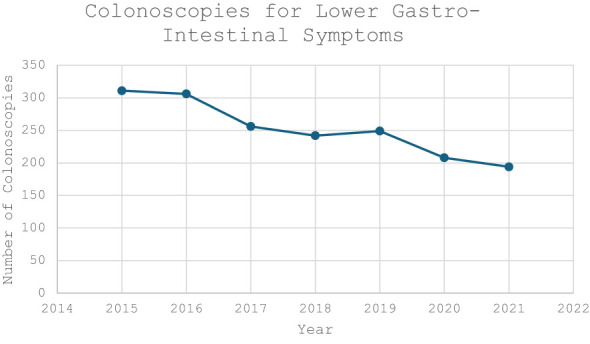
Volume of Colonoscopies performed to investigate lower gastrointestinal symptoms during the study period (2015-2021).

The median age in the screening group was 65 years (IQR 62-67) and 64 years in the symptomatic group (IQR 62-68). There was a male predominance in both the screening (n=1,536, 58.1%) and symptomatic cohorts (n=944, 53.4%) (**Table 1**). There was a trend towards an increasing volume of colonoscopies performed per year in the screening group compared to a gradual decrease in the symptomatic cohort (**Figures 1**, **2**).

**Table 1:** Comparison of lesion detection in the screening and symptomatic cohorts.

	Screening (n=2,640)	Symptomatic (n=1,691)	*p value*
**Age (median, IQR)**	65 (62-67)	64 (62-68).	*-*
**Gender (male)**	58.1% (n=1,536)	53.4% (n=944)	*-*
**Adenoma Detection Rate (ADR)**	56% (n=1477)	26.3% (n=465)	*0.01*
**Sessile Serrated Lesions Detection Rate (SSLDR)**	10.4% (n=275)	8.1% (n=143)	*0.05*
**High Risk Finding**	21.3% (n=562)	7.5% (133)	*<0.001*
**Colorectal Cancer**	4.7% (n=125)	0.9% (n=16)	*<0.001*

### Endoscopic data

In terms of diagnostic yield of neoplasia, there were significant differences in both the adenoma detection rate (ADR) (56% vs 26.3%, p<0.01) and the sessile serrated lesion detection rate (SSLDR) (10.4% vs. 8.1%, p=0.05) in the screening cohort compared to the symptomatic group. High risk findings were more frequently detected in the screening cohort (21.3% vs. 7.5%, p<0.01). Colorectal cancer was detected in 4.7% of FIT +ve colonoscopies compared to 0.9% in those with LGIS (p=<0.001) (**Table 1**). Higher rates of excellent/good bowel preparation was noted in the screening group (83.5% vs. 71.3%, p<0.01) as well as increased use of hyoscine butylbromide (19.2% vs. 3.9%, p<0.05).

### Positive predictive value of symptoms

Overall, lower gastrointestinal symptoms were poor predictors of neoplasia and high-risk findings. Sub-group analysis identified the highest diagnostic yield within the ‘anaemia’ (ADR 24.1%, SSLDR 30.8%, high risk findings 19.5%) and ‘PR bleeding’ (ADR 27.3%, SSLDR 22.4%, high risk finding 27.8%) groups (**Table 2**). The positive predictive values for colorectal cancer ranged from 0.3-2.5% and 0.5-2.5% respectively (**Table 3**).

**Table 2:** Prevalence of neoplasia by lower gastrointestinal symptoms.

Symptom	Adenoma (n=465)	Sessile Serrated Lesion (n=143)	High Risk Finding (n=133)
**Abdominal Pain**	15.3% (71)	12.6% (18)	12% (16)
**Altered Bowel Habit**	10.1% (47)	9.1% (13)	9.8% (13)
**Anaemia**	24.1% (112)	30.8% (44)	19.5% (26)
**Constipation**	7.1% (33)	5.6% (8)	7.5% (10)
**Diarrhoea**	13.5% (63)	12.6% (18)	15.8% (21)
**Bleeding Per Rectum**	27.3% (127)	22.4% (32)	27.8% (37)
**Weight Loss**	3.2% (15)	1.4% (2)	4.5% (6)

**Table 3:** Prevalence of symptoms (n, %), cases of colorectal cancer (n, %) (total n = 16), and Positive predictive values (PPVs) (%) with regard to symptom type (n = 1,767).

Symptom	Present (%)	CRC Cases	PPV CRC
**Altered Bowel Habit**	172 (9.7%)	4 (25%)	2.3%
**Anaemia**	410 (23.2%)	1 (6.25%)	0.5%
**Diarrhoea**	287 (16.2%)	3 (18.75%)	1.1%
**PR Bleeding**	419 (23.7%)	5 (31.25%)	1.2%
**Weight Loss**	80 (4.5%)	2 (12.5%)	2.5%

## Discussion

This retrospective review compared the neoplasia detection in an organised population based colorectal cancer screening cohort with an age-matched cohort of patients referred for investigation of lower gastrointestinal symptoms since the inception of the BowelScreen programme. Although, the CRC screening programme has been in operation in Ireland for almost a decade, the most recent report indicates a poor uptake rate of 41.9%, which is below the minimum European recommendation of >45% (13) and compares poorly with other European FIT based programmes (52.4 - 72.3%) (14, 15). While this may, in part, represent a reluctance of some apparently healthy individuals to participate in screening, it also suggests a poor societal knowledge and appreciation of the benefits of bowel screening, and a failure of health promotion by health practitioners.

Colorectal cancer screening programmes aim to reduce the number of late presentations with advanced disease and, importantly, overall CRC-related mortality. This, however, results in an increase in demand for colonoscopy services which steadily become overwhelmed, causing delays in colonoscopy for FIT-positive screening participants. As colonoscopy is an invasive, costly and limited resource, it is important to accurately identify the most at-risk cohort within a population to whom screening should be targeted, and optimise the resources available for the most effective benefit. Colonoscopy following a FIT positive result in an average risk screening population has been shown to reduce colorectal cancer incidence and mortality (16–18).

FIT can also be employed to stratify symptomatic patients, with a lower threshold of 10µg/g as a triage test for suspected colorectal cancer in both low and high risk symptoms, used to determine whether further investigation is required (19, 20). FIT testing prior to colonoscopy in order to prioritise high risk patients is now supported by guidelines (21). Despite this, patients presenting with lower gastrointestinal symptoms, in particular those falling within the age range of the screening programme, continue to be referred for colonoscopy. In our study, 1,761 colonoscopies were performed on symptomatic individuals in the 60-70 year age group, representing 21.8% of all colonoscopies performed on this age group during the 7 year study period. Perhaps reassuringly, we do note a gradual decrease in the proportion of colonoscopies performed in this age group for symptom assessment during the study period, perhaps reflecting an increasing use of non-invasive testing (22), notwithstanding the effects of the recent global COVID-19 pandemic on access to endoscopic services.

This study demonstrates the significant improvement in neoplasia detection between colonoscopy after FIT based triage within a screening programme and colonoscopy for symptom investigation alone. Risk prediction models for CRC including FIT have previously shown promise in identifying symptomatic individuals most at risk of colorectal neoplasia (23). The adenoma detection rate of 26.3% recorded in the symptomatic group is likely representative of the high quality colonoscopy performed in this group. Nonetheless, there was significant variance within this heterogenous group with ADRs ranging from 3.2%-30.8% based on individual symptoms (**Table 2**), and it is significantly lower than the 56% ADR recorded in the screening cohort (p<0.01). The marginal improvement in SSL detection, although still significant, most likely reflects the relatively poor performance of FIT in SSL detection overall (24). While the detection and resection of adenomatous polyps is an important component of colorectal cancer prevention, it is now understood that the greatest benefit lies in the detection and resection of higher risk, or advanced colorectal polyps (≥1cm) with or without dysplasia. There was a notable difference in the detection of high risk findings between the groups, with 21.3% in the screening cohort compared to just 8.1% in the symptomatic cohort (p<0.01). Most importantly in this age group, FIT outperformed symptoms alone in the detection of colorectal cancer (4.7% vs. 0.9%, p<0.001). Symptoms alone were poor predictors of CRC with a maximal PPV of 2.5% within the symptomatic group. While lower gastrointestinal symptoms should not be ignored, they can be difficult to interpret and are often over investigated (25). An initial triage with FIT can identify higher risk individuals within this heterogeneous groups for whom colonoscopy is warranted (26, 27). Furthermore, inclusion in an organised screening programme with interval repeat FIT testing provides ongoing surveillance which can determine further appropriate investigation (28).

As a dedicated, nurse specialist led service, the CRC screening programme offers efficient triage and education to participants prior to colonoscopy. This is of particular benefit in this age-group, who are more likely to be predisposed to constipation (29), and who may benefit from additional bowel preparation. This is reflected in our study with high rates of excellent/good bowel preparation in the screening group (83.5% vs. 71.3%, p<0.01), with no procedure failed due to poor or insufficient bowel preparation. This facilitates high quality index procedures which undoubtedly has an effect on lesion detection. Symptomatic patients do not receive the same quality of pre-procedure education and it is therefore perhaps not surprising that the quality of bowel preparation is suboptimal in this group, with a 2.3% rate of failed procedures due to poor bowel preparation requiring repeat procedures. Admittedly, repeat procedures in this group might result in a marginal increase in diagnostic yield, however at the significant cost of a repeat colonoscopy.

This study reports the varying practices in use of hyoscine butylbromide between the groups. Diagnostic adjuncts including narrow band imaging (30) (NBI) and distal attachment devices (e.g. Endocuff Vision) (31, 32) which have been shown to increase lesion detection, are more frequently employed in a screening setting. The use of hyoscine butylbromide has, more recently, been associated with an improvement in both adenoma and sessile serrated lesion detection (33, 34). This study reports a significant difference in practice with increased use in the screening cohort (19.2% vs. 3.9%, p<0.05).

There are a number of limitations to this study that should be acknowledged, primarily the retrospective nature of the study which may give rise to recall or selection bias. The prospectively maintained BowelScreen database, and electronic endoscopic records were carefully reviewed by 3 independent researchers to minimise this effect. The screening colonoscopies were all completed by high performing colonoscopists (>300 colonoscopies/year, ADR >45%) while the non-screening colonoscopies were performed by senior endoscopists as well as trainee endoscopists. While no minimum annual volume of colonoscopy is required of them, a minimum ADR of >15% is required and audited by the national quality assurance programme. This could arguably result in better neoplasia detection in the screening group, however this cannot fully explain the significant differences between the groups. Further matching between the groups beyond age was not performed, however a similar male predominance was noted in both groups (58.1% & 53.4%). Family history and other dietary and lifestyle factors were not included in this study. The inclusion of risk factors to develop tailored CRC screening programmes holds promise, although a recent study combining a risk model including risk factors with FIT did not increase the yield of advanced neoplasia in a screening cohort (35). A multicentre study assessing the diagnostic yield of personalised vs. uniform screening is ongoing (36).

There is an inherent difficulty in comparing these two groups who present for colonoscopy from different referral pathways which could, of course, influence the outcomes reported. On one hand, FIT is known to have a high sensitivity for adenoma detection, while on the other it could be argued that symptomatic patients in this age group might be expected to have a higher pre-test probability for pathology. Nevertheless, this is a reflection of current real life clinical practices. Index only colonoscopies were included in this study to reduce the effect of surveillance bias.

In the context of poor uptake of the organised screening programme, this study reflects an over utilisation of colonoscopy services prompted by symptom assessment. The focus, in this age group, should be on directing individuals towards the organised screening programme for a FIT based approach.

## Conclusion

Colonoscopy is a limited resource which should be targeted at higher risk individuals either through an organised FIT based colorectal cancer screening, or by way of non-invasive biomarker testing in a symptomatic cohort, for which cut-off points should be agreed. Patients within the screening age group should be preferentially referred to the BowelScreen programme, to facilitate appropriate triage as well as preparation and patient education pre colonoscopy, and to confer the benefits of screening colonoscopy (higher quality, better bowel preparation, increased use of diagnostic adjuncts) for the participant. Failing this, non-invasive stool testing should be performed to identify low risk patients in whom colonoscopy can be avoided, and reserved for higher risk individuals. This should build further capacity and facilitate the expansion of the screening programme to include a younger cohort.

## Data availability statement

The raw data supporting the conclusions of this article will be made available by the authors, without undue reservation.

## Ethics statement

Ethical approval was not required for the studies on humans in accordance with the local legislation and institutional requirements because only commercially available established cell lines were used.

## Author contributions

NO’M: Conceptualization, Data curation, Writing – original draft, Writing – review & editing. RS: Writing – review & editing. JD: Writing – review & editing. BN: Data curation, Writing – review & editing. PG: Data curation, Writing – review & editing. LK: Writing – review & editing. MM: Data curation, Writing – review & editing. EJ: Writing – review & editing. OC: Data curation, Writing – review & editing. GH: Conceptualization, Methodology, Writing – review & editing. GD: Conceptualization, Formal Analysis, Methodology, Supervision, Writing – review & editing.
